# Perceived Stress Latent Factors and the Burnout Subtypes: A Structural Model in Dental Students

**DOI:** 10.1371/journal.pone.0099765

**Published:** 2014-06-13

**Authors:** Jesús Montero-Marín, Marcelo Marcos Piva Demarzo, Lexine Stapinski, Margarita Gili, Javier García-Campayo

**Affiliations:** 1 Faculty of Health and Sport Sciences, Huesca, Spain; 2 Department of Preventive Medicine, Universidade Federal de São Paulo (UNIFESP), São Paulo, Brasil; 3 School of Social and Community Medicine, University of Bristol, Bristol, United Kingdom; 4 Centre for Research Excellence in Mental Health and Sustance Use, University of New South Wales, Sidney, Australia; 5 Institut Universitari d'Investigació en Ciències de la Salut (IUNICS), University of Balearic Islands, Mallorca, Spain; 6 Miguel Servet University Hospital, University of Zaragoza, Zaragoza, Spain; 7 Primary Care Prevention and Health Promotion Research Network (RedIAPP), Zaragoza, Spain; Federal University of Rio de Janeiro, Brazil

## Abstract

**Background:**

Students of health-professions suffer high levels of stress and burnout. The aim of the present study was to evaluate the relationship between perceived stress latent factors (‘tenseness’ and ‘frustration’) and the features (‘overload’, ‘lack of development’ and ‘neglect’) of the three burnout subtypes (‘frenetic’, ‘under-challenged’ and ‘worn-out’, respectively), in a sample of Spanish dental students.

**Methods:**

The study employed a cross-sectional design. A sample of Spanish dental students (n = 314) completed the ‘Perceived Stress Questionnaire’ and the ‘Burnout Clinical Subtype Questionnaire Student Survey’. The associations among variables were observed by means of structural equation modelling using the unweighted least squares method from polychoric correlations.

**Results:**

Strong associations among perceived stress factors and the burnout characteristics were observed, although a distinct pattern of relations was observed for each burnout subtype. The ‘overload’ was moderately and positively associated with both ‘tenseness’ (0.45), and ‘frustration’ (0.38) dimensions of perceived stress; the ‘lack of development’ was positively associated with the ‘frustration’ dimension (0.72), but negatively associated with ‘tenseness’ (−0.69); the ‘neglect’ showed a weaker positive associated with ‘frustration’ (0.41), and a small negative association with ‘tenseness’ (−0.20). The model was a very good fit to the data (GFI  =  0.96; RSMR  =  0.07; AGFI = 0.96; NFI = 0.95; RFI = 0.95).

**Conclusions:**

The stress factors of ‘frustration’ and ‘tenseness’ seems to be related in a distinct way to the burnout subtypes in Spanish dental students. This finding suggests that intervention programs specifically tailored to these subtypes may be a promising future direction.

## Introduction

Students of health-professions suffer high levels of stress and burnout while completing their undergraduate study, with negative consequences for their personal and professional life [1], [2]. The training period to become a dentist involves considerable sources of potential stress, such as limited free time, examinations and supervisors' clinical requirements [3]–[5]. As a result, the prevalence of burnout among dental students and newly graduated dentists is alarmingly high. The incidence of burnout is of considerable relevance to the profession given the associated impact on academic and professional performance, use of medication, and risk of dropping out of the course and career [6], [7].

High levels of perceived stress occurs when environmental demands overwhelm an individual's resources and threaten his personal well-being [8]. In dental students, a hierarchical bi-factor structure of perceived stress has been identified [9]. The ‘tenseness’ dimension is the perception that one is hurried, with too many things to do, and is the consequence of external demands. The ‘frustration’ dimension describes the negative affective aspect of stress, and is characterised by feelings of discouragement, joylessness and worry [10], [11]. Related to perceived stress, burnout syndrome is an inability to cope with chronic occupational stress and is an attempt to adapt to or protect oneself from it [12]. This syndrome has been characterised by a state of exhaustion, cynicism and inefficacy [13]. Exhaustion is the feeling of not being able to offer any more of oneself at an emotional level; cynicism represents a distant attitude towards work, those served by it and other colleagues; and inefficacy is the feeling of not performing tasks adequately or being incompetent. The presence of burnout syndrome is related to poorer perceived health and high rates of somatic comorbidity [14], [15].

A more comprehensive definition of burnout has recently been proposed to differentiate three different clinical subtypes of the syndrome [16]. The ‘frenetic’ type of burnout is characterized by ‘overload’, the perception of jeopardising one's health to pursue worthwhile results, and is highly associated with exhaustion. The ‘underchallenged’ type of burnout is characterized by ‘lack of development’, defined as the perception of a lack of personal growth, together with the desire for a more rewarding occupation that better corresponds to one's abilities, and is most strongly associated with cynicism. The ‘worn-out’ type of burnout is characterised by ‘neglect’, defined as a inattentive and careless response to responsibilities, and is closely associated with inefficacy [17]. The dimensions of ‘overload’, ‘lack of development’ and ‘neglect’ show great explanatory power over the classical burnout definition, while having a significant ability to distinguish the different profiles [18].

The burnout subtypes can be ordered according to level of task dedication, which affects the way individuals manage the feelings of distress. Altering level of task dedication may be a way for individuals to exert some control over the balance between effort invested and rewards gained [16]. The most dedicated profile is the ‘frenetic’, with its active coping style, while the least dedicated is the ‘worn-out’, because of its passive style. Therefore, the degree of task dedication acts as a classification criterion of the burnout typology. This classification criterion is consistent with the idea of a developmental transition between the different burnout profiles driven by changes in dedication, from the more dedicated to less [16]–[19]. Each stage of burnout may correspond to a different pattern of perceived stress as a result of differing levels of tasks dedication.

There are no studies assessing potential relationships among perceived stress factors and burnout clinical subtypes in dental students, or health professionals in general, despite the expected causal paths among them as reviewed above. Therefore, the main goal of the present study was to evaluate the association among perceived stress factors (‘tenseness’ and ‘frustration’) and the main burnout subtypes features (‘overload’, ‘lack of development’ and ‘neglect’) in dental students.

We examined the theory-driven hypotheses related to potential causal paths among constructs, with a view to informing the develoment of tailored early intervention approached. Covariance structures, not used before in this area of research, were chosen to evaluate the following assumptions: the latent factors of perceived stress (‘tenseness’ and ‘frustration’) and the latent factors of the burnout subtypes (‘overload’, ‘lack of development’ and ‘neglect’) are strongly related (Hypothesis 1), although with different patterns for each burnout profile, because of their distinct features (Hypothesis 2). It is expected that 'tenseness' affects most to 'overload', and 'frustration' to 'lack of development'. According to the burnout subtypes developmental theory [19], we also expected important links between the burnout profiles, routed from most to least level of dedication (Hypothesis 3).

## Methods

### Study design

We used a cross-sectional design. Participants completed a paper-and-pencil battery of self-assessment instruments.

### Participants

The population consisted of Spanish dental students enrolled at the Huesca (N_H_ = 136) and Santiago de Compostela (N_S_ = 242) campuses during the 2010–2011 academic term. Sample size was estimated according to the recommended 10∶1 ratio of the number of participants to the number of the test items [20]. All enrolled students were sent the survey and 83.1% responded resulting in a sample of n = 314 participants. The subjects did not receive any payment or credit compensation in return for participation in the study.

### Procedure and ethics statement

A clinical psychologist trained two research assistants to administer the questionnaires. The first page of the protocol identified the objectives of the study, the prospective participants, the potential benefits and risks and the confidentiality of the data, so that each participant provided written informed consent before completing the survey. The research assistants administered the survey two weeks before the final exam period, in May 2011. After completion, the questionnaires were collected and kept in a sealed envelope to ensure the participants' anonymity. The study protocol was approved by the Ethical Review Board of Aragon, Spain. This study followed Helsinki Convention norms and later modifications, the Declaration of Madrid of the World Psychiatric Association and the uniform requirements for manuscripts submitted to Bio-medical journals.

### Measures

#### Socio-demographic data

We collected information on age, sex, stable relationship (‘yes’, ‘no’), children (‘yes’, ‘no’), scholarship (‘yes’, ‘no’), campus (‘Huesca’, ‘Santiago’), weekly studying hours, failed subjects in the previous semester (‘yes’, ‘no’), job (‘yes’, ‘no’), and year of study (‘first’, ‘second’, ‘third’, ‘fourth’, ‘fifth’).

#### Perceived Stress Questionnaire (PSQ)

We used the Spanish version of the PSQ recent form, which addresses the last 30 days [11], [21]. This questionnaire has been validated for dental student samples [9], and comprised twenty-four items, equally distributed between the two perceived stress dimensions ‘tenseness’ (e.g., ’You have too many things to do’) and ‘frustration’ (e.g., ’You feel discouraged’). The participants showed their agreement with the items on a 4-point Likert scale with responses ranged from 1 (‘almost never’) to 4 (‘almost always). The scores for each dimension are calculated by a linear algorithm and ranged from 0 to 1. The factorial and convergent validity, as well as the internal consistency, have been adequate within the study’s target population [9]. The PSQ has been used in research, demonstrating good predictive validity for stress-related diseases, such as ulcerative colitis [22], [23]. It is also correlated with somatic symptoms of psychological origin and with the presence of psychopathological diseases as evaluated by SPPI psychiatric interview [21].

#### Burnout Clinical Subtype Questionnaire - Student Survey (BCSQ-12-SS)

The participants also completed the Spanish version of the ’Burnout Clinical Subtype Questionnaire’ [17]. We used the brief BCSQ-12-SS, which had been adapted and validated for student respondents [24]. The adaptation transformed the original scale items that referred to employment activities into 12 items that referred to student activities. The BCSQ-12-SS items are evenly distributed among the dimensions of ‘overload’ (e.g., ’I think that I dedicate more effort to my studies than I should for my health’), ‘lack of development’ (e.g., ’I would like to be studying material that challenges my abilities more’) and ‘neglect’ (e.g., ’When my studies don’t turn out as well as they should, I stop trying’). The participants rated their agreement with the items on a 7-point Likert scale with responses that ranged from 1 (‘completely disagree’) to 7 (‘completely agree’). The scores for each dimension were calculated by a linear algorithm and ranged from 0 to 1. The factorial structure, internal consistency and convergent validity have been deemed adequate for workers [17] and for the study's target population more specifically [24].

### Data analyses

Analyses were conducting use the SPSS-19.0, FACTOR-9.02 and AMOS-7.0 statistical packages. Means, standard deviations, skewness, kurtosis and item-rest coefficients were calculated to evaluate the performance of the PSQ and BCSQ-12-SS items. We also estimated Mardia's coefficients to assess the multivariate normality distribution of them [25]. Polychoric correlations are advised for structural equation modelling (SEM) when the distributions of ordinal items are asymmetric, with excess of kurtosis or high item-rest coefficients [26]. Thus, polychoric correlation matrices with regard to the PSQ and BCSQ-12-SS items were estimated. We verified the adequacy of the correlation matrices, assessing the determinant, the Kaiser-Meyer-Olkin (KMO) index and the Barlett's test of sphericity [27].

An unweighted least squares (ULS) was the method used for developing covariance structures [28]. ULS estimation does not provide inferential procedures for assessing model data fit based on the χ^2^ distribution, but it does not require any distributional assumptions; is quite robust and usually converges because of its efficiency in terms of computation. Moreover, in complex solutions tends to provide less biased estimates of the true parameter values than classical methods [29]; is an appropriate choice for moderately sized samples; shows good performance when working with polychoric matrices; tends to provide accurate estimates even with large models; and seems to provide better estimates than more complex procedures [30]–[32].

**Figure 1:**
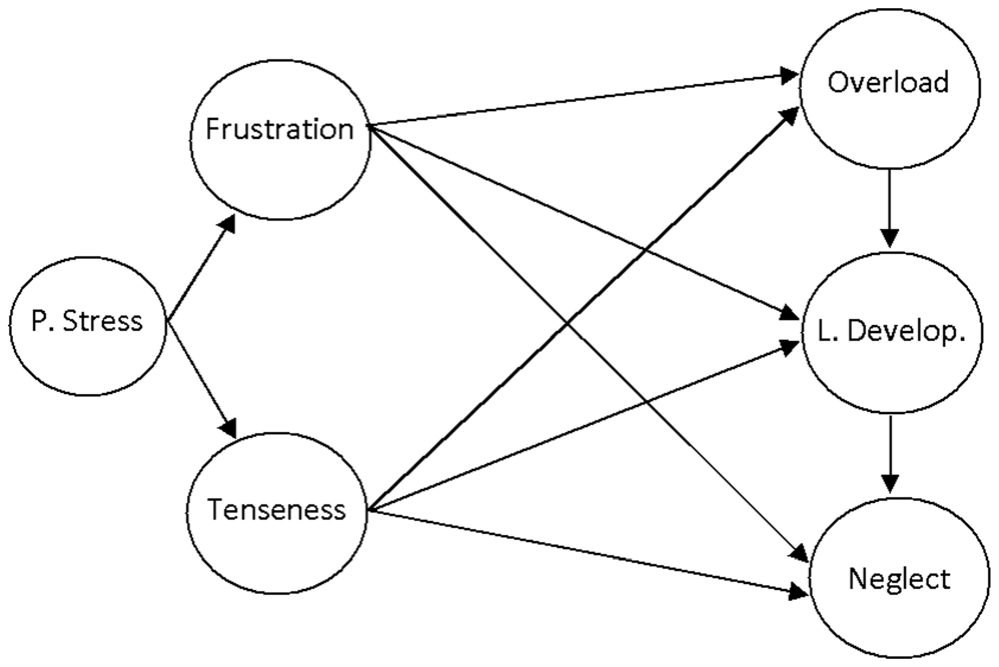
Hypothetical structural model.

Firstly, we applied ULS from polychoric correlation matrices to test the fit of the PSQ and BCSQ-12-SS measurement models by CFA. Secondly, we used structural equations modelling to evaluate the empirical links between the PSQ and BCSQ-12-SS dimensions. To evaluate model fit to the data, we examined the gamma goodness of fit index (GFI), the adjusted goodness-of-fit index (AGFI), the root mean square of the standardized residuals (RMSR), the normed fit index (NFI) and the Bollen's relative fit index (RFI). GFI and AGFI refer to explained variance and values >0.90 are considered acceptable [33]. SRMR is the standardized difference between the observed and the predicted covariance, indicating a good fit for values <0.08 [34]. NFI measures the proportional reduction in the adjustment function when going from null to the proposed model and is considered acceptable when >0.90 [35]. RFI takes into account the discrepancy for the model evaluated and for the baseline model, and is very good close to 1 [36]. All of these indices are valid for ULS procedure. Taken together, they provide a reliable evaluation of the solution and additional information regarding absolute and incremental model-data fit assessment. The factor weights, the explained variance and the association between latent factors, all of which standardized, were also taken into account to examine the pattern of relationships. We can observe the hypothetical structural equations model in Figure 1.

## Results

In order to adhere to standards for data availability, the authors state that all materials used to produce the results in this paper will be made available upon request. This includes [37]: 1.- The list of documents and data files that are needed in order for replication to be possible, 2.- A detailed list of what will be provided by the authors, and 3.- What steps, and in what sequence, the interested researchers need to take in order for this data to be made available. In addition, authors will post these materials on the group's website [38].

### Characteristics of participants

The participants were European adults between the ages of 18 and 41 (Mean = 22.05; SD = 3.57). Table 1 shows the characteristics of the participants. No differences were found in response rate based on sex [‘men’ = 81.4% vs. ‘women’ = 83.8%; χ^2^_(df = 1)_ = 0.31; p = 0.576], campus [‘Huesca’ = 87.5% vs. ‘Santiago’ = 80.6%; χ^2^_(df = 1)_ = 2.97; p = 0.085] or age [‘participants’ Mean = 22.05; SD = 3.57 vs. ‘non-participants’ Mean = 22.34; SD = 3.83; t = 0.60_(df = 375)_; p = 0.551].

**Table 1:** Characteristics of the participants (n = 314).

Age, Md (SD)	22.05 (3.75)
Sex, females (%)	222 (70.7)
Stable relationship, no (%)	158 (50.5)
Children, none (%)	300 (95.5)
Scholarship, no (%)	199 (63.4)
Campus, Santiago (%)	195 (62.1)
Weekly studying hours, Md (SD)	37.27 (17.52)
Failed subjects, no (%)	212 (67.9)
Job, no (%)	266 (84.7)
First year of study (%)	62 (19.8)
Second (%)	63 (20.0)
Third (%)	60 (19.1)
Fourth (%)	69 (22.0)
Fifth (%)	60 (19.1)

Md  =  Mean; SD  =  Standard Deviation.

Number and percentage (%).

### Descriptive statistics

Descriptive statistics for the PSQ items can be seen in Table 2. The items n° 5 (‘you feel lonely or isolated’) and n° 6 (‘you find yourself in situations of conflict’) presented skewness values >1.00. Otherwise, the items n° 10 (‘you feel calm’), n° 19 (‘you are under pressure from other people’), n° 22 (‘you are afraid for the future’) and n° 30 (‘you feel under pressure from deadlines’), showed kurtosis values <−1.00. Mardia’s multivariate skewness and kurtosis coefficients were 132.70 (p = 1.00) and 1,040.71 (p<0.001), respectively. The item-rest values were very high and positive, ranged from 0.41 (item n° 11, ‘you have too many decisions to make’) to 0.76 (item n° 28, ‘you feel loaded down with responsibility’). Table 3 shows the descriptive for the BCSQ-12-SS items. The items n° 3 (‘When the results of my studies are not good at all, I stop making an effort’), n° 6 (‘I give up in response to an obstacle in my studies’), n° 9 (‘I give up when faced with any difficulty in my tasks as a student’) and n° 12 (‘When the effort invested in studying is not enough, I give up’) presented skewness values >1.00. The item n° 6 also showed a kurtosis value >1.00. Mardia's multivariate skewness and kurtosis coefficients were 31.47 (p = 1.00) and 232.58 (p<0.001), respectively. The item-rest values were very high and positive, ranged from 0.53 (item n° 2, ‘I would like to study something else that would be more challenging to my abilities’) to 0.78 (item n° 7, ‘I am endangering my health in pursuing good results in my studies’). All of these results indicated the need to use polychoric correlations for SEM.

**Table 2:** Descriptives of the ‘Perceived Stress Questionnaire’ (PSQ).

Items	Mn	SD	Skew	Kurt	r_i(t-i)_
*Tenseness (0-1)*	*0.56*	*0.22*			
2. You feel that too many demands are being made on you	2.65	0.94	−0.01	−0.94	0.56
4. You have too many things to do	3.11	0.86	−0.57	−0.59	0.64
8. You feel tired	2.83	0.90	−0.17	−0.93	0.66
11. You have too many decisions to make	2.59	0.80	0.15	−0.53	0.41
14. You feel tense	2.38	0.91	0.21	−0.74	0.69
16. You feel you're in a hurry	2.73	0.94	−0.23	−0.85	0.68
18. You have many worries	2.73	0.93	−0.14	−0.92	0.74
26. You feel mentally exhausted	2.52	0.96	0.10	−0.94	0.74
27. You have trouble relaxing	2.27	1.03	0.37	−0.97	0.67
28. You feel loaded down with responsibility	2.53	0.94	0.09	−0.90	0.76
29. You have enough time for yourself (R)	2.97	0.89	−0.52	−0.52	0.56
30. You feel under pressure from deadlines	2.80	0.95	−0.19	−1.01	0.64
*Frustration (0-1)*	*0.35*	*0.20*			
5. You feel lonely or isolated	1.56	0.81	1.35	1.05	0.47
7. You feel you're doing things you really like (R)	1.72	0.77	0.81	0.04	0.43
9. You fear you may not manage to attain your goals	2.34	0.95	0.32	−0.79	0.60
10. You feel calm (R)	2.58	1.01	−0.13	−1.05	0.63
12. You feel frustrated	1.80	0.83	0.86	0.19	0.67
13. You are full of energy (R)	2.63	0.91	−0.19	−0.74	0.65
17. You feel safe and protected (R)	2.29	0.91	<0.01	−0.95	0.59
20. You feel discouraged	1.92	0.84	0.76	0.11	0.72
21. You enjoy yourself (R)	2.12	0.95	0.38	−0.85	0.62
23. You feel you're doing things because you have to (…)	1.98	0.89	0.63	−0.34	0.49
24. You feel criticized or judged	1.82	0.86	0.78	−0.22	0.53
25. You are light hearted (R)	1.97	0.80	0.44	−0.38	0.71

The numbers of the items are according to the original 30-items PSQ. R  =  reversed. Mn  =  mean. SD  =  standard deviation. Skew  =  skewness. Kurt  =  kurtosis. r_i(t-i)_  =  item-rest correlation.

**Table 3:** Descriptives of the ‘Burnout Clinical Subtype Questionnaire’ (BCSQ-12-SS).

Items	Mn	SD	skew	kurt	r_i(t-i)_
*Overload (0-1)*	*0.39*	*0.24*			
1. I think I invest more than is healthy in my commitment to my studies	4.07	1.59	0.11	−0.50	0.58
4. I neglect my personal life due to pursuing great objectives in studying	3.26	1.77	0.40	−0.73	0.68
7. I am endangering my health in pursuing good results in my studies	2.98	1.84	0.64	−0.56	0.78
10. I ignore my own needs to satisfy the requirements of my studies	2.98	1.75	0.68	−0.36	0.73
*Lack of development (0-1)*	*0.24*	*0.20*			
2. I would like to study something else that would be more challenging to my abilities	2.73	1.65	0.64	−0.43	0.53
5. I feel that my current studies are hampering the development of my abilities	2.32	1.35	0.90	0.35	0.66
8. I would like to study something else in which I could better develop my talent	2.41	1.61	0.92	−0.13	0.72
11. My studies do not provide me with opportunities to develop my abilities	2.37	1.45	0.88	0.01	0.64
*Neglect (0-1)*	*0.18*	*0.17*			
3. When the results of my studies are not good at all, I stop making an effort	2.25	1.41	1.13	0.91	0.59
6. I give up in response to an obstacle in my studies	2.14	1.35	1.33	1.64	0.63
9. I give up when faced with any difficulty in my tasks as a student	1.85	1.06	1.07	0.30	0.70
12. When the effort invested in studying is not enough, I give up	2.03	1.22	1.09	0.56	0.66

The numbers of the items are according to the original 12-items ‘Burnout Clinical Subtype Questionnaire’ (BCSQ-12-SS).

Mn  =  mean. SD  =  standard deviation. Skew  =  skewness. Kurt  =  kurtosis. r_i(t-i)_  =  item-rest correlation.

### Measurement models

The polychoric correlation matrix of the PSQ items revealed that 78.6% coefficients out of the diagonal were ≥0.30. The determinant was <0.001, KMO test had a value of 0.95 and Bartlett's statistic was 4,780.50 (df = 435; p<0.001). Thus, the behavior of the PSQ items allowed factorial analysis to be performed with guarantees. The PSQ bi-factor structure presented good fit indices using CFA, without correlations among the error terms (GFI = 0.98; RSMR = 0.06; AGFI = 0.98; NFI = 0.98; RFI = 0.98). The items demonstrated high loadings on their corresponding latent factor (‘frustration’ and ‘tenseness’ ranges  = 0.42 to 0.81 and  = 0.38 to 0.85, respectively), indicating that the PSQ measurement model was adequate.

The polychoric correlation matrix of the BCSQ-12-SS items showed that 57.6% coefficients were ≥0.30. The determinant was  = 0.004, KMO  = 0.82 and Bartlett's statistic was 1,675.00 (df = 66; p<0.001). Thus, the distribution of the BCSQ-12-SS items supported the subsequent factorial analysis adequately. The BCSQ-12-SS tri-factor structure presented adequate fit using CFA, without correlations among the errors (GFI = 0.98; RSMR = 0.07; AGFI = 0.96; NFI = 0.96; RFI = 0.95). The item loadings were high (‘overload’, ‘lack of development’ and ‘neglect’ ranged from 0.51 to 0.86, from 0.52 to 0.91 and from 0.65 to 0.84, respectively), indicating that the BCSQ-12-SS measurement model was correct.

### Structural equations model

**Figure 2:**
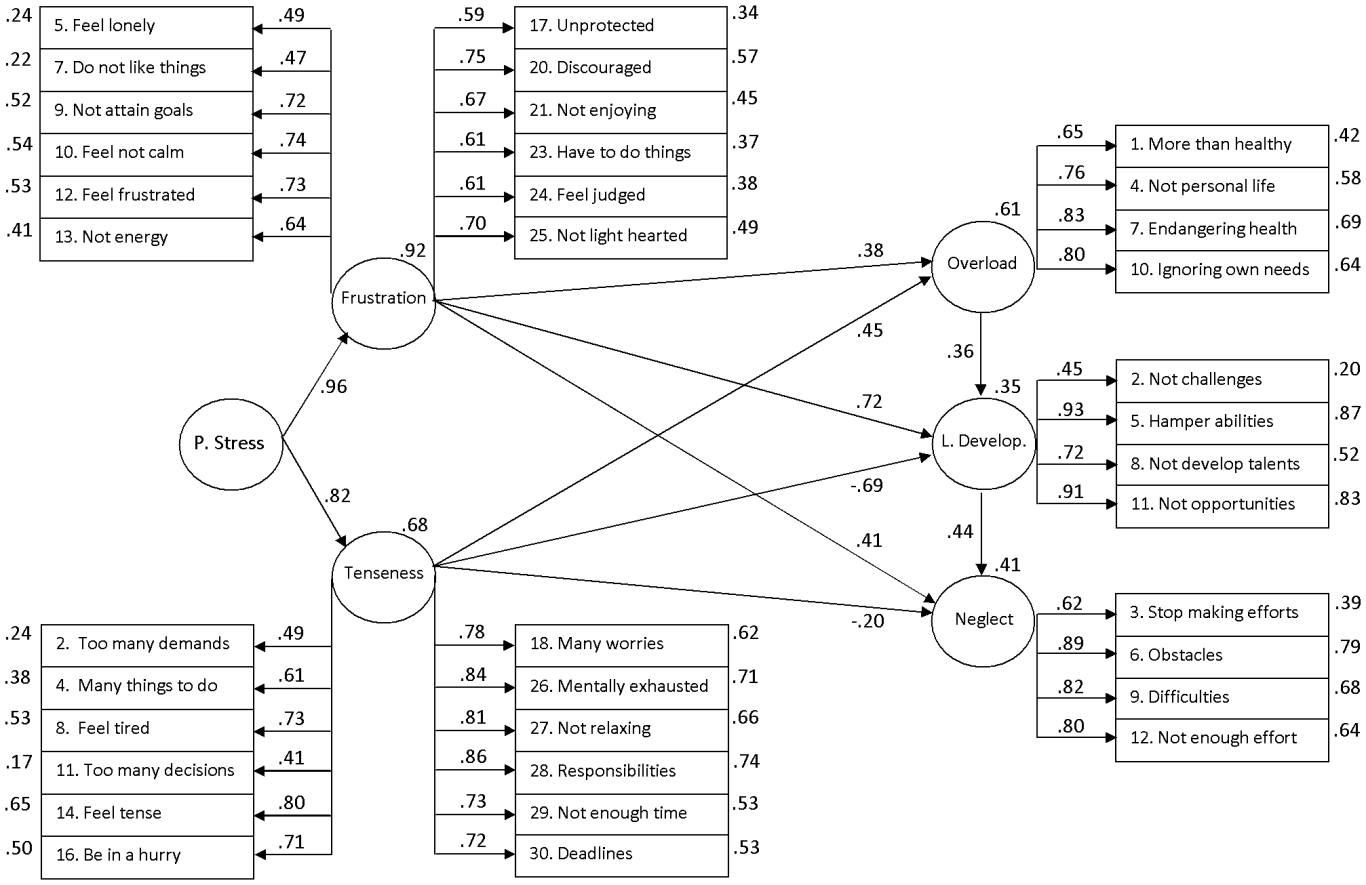
Pattern of relationships between the latent factors of the PSQ hierarchical bi-factor structure and the burnout types. Standardized estimates from SEM. Circles represent latent constructs and rectangles are observable variables. Factor weights are over the arrows and the percentage of explained variance over the circles and the boxes.

Figure 2 shows the pattern of relationships among the perceived stress and the burnout types. Variance in both stress and burnout latent factors was highly explained, with R^2^ values from 0.35 (‘lack of development’) to 0.92 (‘frustration’), therefore, the explanatory power of the model was high. On the other hand, the relationships among the constructs were remarkable. In the case of the ‘overload’ dimension, moderately high and positive associations were observed with the perceived stress factors ‘frustration’ (0.38) and ‘tenseness’ (0.45). For the ‘lack of development’ dimension, the association with ‘frustration’ was high and positive (0.72), but high and negative with ‘tenseness’ (−0.69). For the ‘neglect’ dimension, associations with ‘frustration’ were moderately high and positive (0.41), but moderately low and negative for ‘tenseness’ (−0.20). The links among the burnout types according to the degree of dedication reached moderately high and positive values (0.36 from ‘overload’ to ‘lack of development’ and 0.44 from ‘lack of development’ to ‘neglect’). All of fit indices were within acceptable limits (GFI = 0.96; RSMR = 0.07; AGFI = 0.96; NFI = 0.95; RFI = 0.95). Adding a link between ‘overload’ and ‘neglect’ showed a standardized value of 0.02 and worsened the model fit (GFI = 0.96; RSMR = 0.08; AGFI = 0.95; NFI = 0.94; RFI = 0.94). Omitting the proposed relationships between the burnout types also worsened the model fit (GFI = 0.95; RSMR = 0.09; AGFI = 0.94; NFI = 0.94; RFI = 0.93). Consequently, the theoretically proposed model showed a reasonably good fit to the data.

## Discussion

This is the first study that has evaluated possible relationships among the perceived stress in dental students [9]–[11], with the typological proposal for burnout syndrome [17], [18], [24]. Other works have indicated the relevance of coping with stress to burnout syndrome [39], [40], but have not studied the influence of specific stress dimensions with regard to the different burnout subtypes. A SEM analysis was computed to assess the pattern of relationships between the perceived stress bi-factor structure and the burnout subtypes. Overall, the data supported our hypotheses. In general, there were strong associations among the perceived stress and burnout constructs, and each burnout type showed a distinctive pattern of associations with perceived stress, being strong between ‘tenseness’ and ‘overload’, and between ‘frustration’ and ‘lack of development’. Furthermore, the features of the burnout sub-types showed important paths along the continuum of task dedication. These findings are consistent with the idea of the development of the burnout syndrome through stages by subtracting implication to protect against perceived stress [19], [41], and may be relevant to develop new treatments and preventive programs on burnout, adjusted by the specific characteristics of dental students.

An important strength for the present study is that it was carried out using a high stress-risk sample [3]–[5] and it was conducted during the period of final exams, a well-known source of stress. Despite students in the first years of college not having contact with patients, they may suffer from other causes of stress, such as limited free time, supervisors’ requirements or examinations [3]–[5]. Inasmuch as burnout is a general inability to cope with chronic stress, not only for dealing with people but because of several sources of distress, and is an attempt to adapt to or protect oneself from it [12], we may suppose that the period of final exams could exacerbate the cumulative symptoms of both stress and burnout, making the results more relevant [42]. Furthermore, an independent researcher supervised the data transcription process to control for errors, and the analysis method respected the true non-linear nature of the variables used. The main limitation of this study is the fact that the cross-sectional design used does not allow us to draw firm conclusions about the etiology of burnout subtypes. This sort of design permits only for the evaluation of relationships among variables at one point in time, and thus can only suggest but not confirm possible causal pathways [43]. On the other hand, the study sample was recruited from two different universities in two Spanish regions and both groups exhibited high and similar response rates, although it is possible that these universities are not representative of the broader population of Spanish dental students. It is also possible that our results among dental students could not be extrapolated to other health professions, as there are major differences among them which warrant specific studies on each group to confirm this.

The participants in the study were young adults, primarily women who did not have children, thus the potential confound of child-rearing stress was avoided. The majority of participants had not received study grants, studied at the University of Santiago, passed all subjects in the previous semester, were not employed and almost half were in a stable relationship. Participants were equally distributed across the five years of study and spent a large amount of time studying per week. In general, responses to the items were in the midrange of the scale, with higher values for ‘frustration’ compared to ’tenseness’, and higher values for ‘overload’ compared with ‘lack of development’ and ‘neglect’. These findings might be explained by considering the stressing although motivating pressure that the students experienced owing to the proximity of final exams [3]–[5], [9]. In general, the nature and behavior of the items, with high and positive item-rest values, as well as the presence of non-linear distributions, recommended the use of ULS method from polychoric correlation matrices. As a result, our findings provide evidence of a perceived stress model together with the different burnout types as a reasonable approximation to the reality studied [44].

The most salient finding of this study was the marked pattern of relationships among the perceived stress factors and the burnout types. Each profile of burnout showed a distinct pattern of association with the perceived stress dimensions, and this may explain some of the differences between them. Firstly, strong and positive links were observed from both ‘tenseness’ and ‘frustration’ to ‘overload’, indicating that the frenetic profile of burnout could be characterized by a large amount of stress, from two different sources. The ‘frenetic’ subtype refers to a category of subjects who are very involved and ambitious and who overload themselves to fulfill the demands of their tasks [19], [41]. This profile of burnout is associated with a disproportionate degree of dedication [17], [45] and could lead to increasing exhaustion levels [19], [41], [46]. Although ‘frustration’ has a prominent importance, we have seen that both ‘tenseness’ and ‘frustration’ are linked to ‘overload’, maybe because of frenetic subtype needs to obtain major successes and achievements. Consequently, to decrease ‘overload’, it seems advisable not only to reduce activation levels but also to fix the aspirations in a realistic and flexible way [40].

Secondly, we noticed high links from ‘tenseness’ to ‘lack of development’, in a negative sense, and from ‘frustration’ to ‘lack of development’, in a positive way, so that the ‘under-challenged’ profile could be suffering stress from only one of the sources referred, although in a high level. This subtype of burnout includes indifferent and bored subjects, who fail to experience personal development in their tasks [19], [41]. Moreover, this profile of burnout is related to boring occupations and large study institutions [17], [45], that may lead to cynical attitudes due to the lack of satisfaction and loss of interest [47], [19], [41]. Paradoxically, although ‘frustration’ increases risk for ‘lack of development’, ‘tenseness’ seems to be a protective factor, maybe because it might facilitate the recuperation of some degree of dedication or accomplishment. So, one method of reducing ‘lack of development’ may be to improve the level of task performance, and thus engagement with work [40].

Similarly, we observed negative associations between ‘tenseness’ and ‘neglect’, and positive links between ‘frustration’ and ‘lack of development’. These relations were smaller than for the previous cases, thus, the ‘worn-out’ profile of burnout may be considered the least stressed subtype. This subtype refers to subjects who present with feelings of lack of control over future outcomes and a sense that their efforts are futile, which ultimately lead to neglect of responsibilities [19], [41]. This profile is negatively influenced by the effect of the organizational structure, in the case of workers, and it is associated with more failed subjects, in the case of students [17], [45]. It could exacerbate the burnout syndrome through passive and ineffective coping strategies when faced with ‘frustration’ [19], [40], [41]. In other words, the abandonment somehow seems to be not free of payment in terms of distress, although it seems to be an attempt to protect from it. Thus, if we want to increase the level of perceived efficacy to reduce neglect, it seems relevant to recover the initial level of investment [48].

Another important finding of this study was the relationships observed among the features of the subtypes of burnout. As we expected, the subtypes were highly linked from most to less level of engagement, something that is consistent with the development progress of the burnout syndrome in general, as it is understood [18]. The progressive decrease in levels of dedication seems to be the response adopted by subjects experiencing burnout to cope with stress [40]. The longitudinal theoretical proposal for the burnout subtypes is a hypothesis which understands the development of the syndrome along the different profiles, from more to less degree of dedication [19], [41]. This hypothesis can explain why some of the more invested and responsible subjects are eventually burned out and why early intervention is so important.

Specific interventions are in particular demand for populations that are highly affected by burnout syndrome, such as dentists [24]. In fact, dental universities and other health students have been advised to incorporate the instruction of stress management skills into their programmes [7]. However, these programs primarily address the ‘tenseness’ latent factor, but tend not to address feelings of ‘frustration’, which may benefit from interventions based on mindfulness and personal values such as Acceptance and Commitment Therapy (ACT) [49]. Taken together, the findings of this study support the tailoring of interventions according to the source and type of stress associated with the distinct burnout profiles. Considering the economic and health implications derived from this knowledge, these findings must be applied at the prevention level, in the education of the students [50].

## Conclusions

We observed that perceived stress factors differ concerning their association to the burnout profiles in dental students. Based on these findings, it is possible that dental students perceive and respond to stress in different ways, balancing ‘tenseness’ and ‘frustration’. It implies that we should identify these response patterns in order to develop effective preventive and therapeutic programs that address the specific characteristics and stress demands associated with the distinct burnout subtypes.
